# A global survey of blood transfusion practices for patients with sickle cell disease

**DOI:** 10.1111/trf.70331

**Published:** 2026-07-15

**Authors:** Jeremy W. Jacobs, Arwa Z. Al‐Riyami, Jeannie Callum, Meghan Delaney, David Daniel, Jenny White, Christopher Bocquet, Mohammed Farouk, Claude Tayou, Chinelo P. Onyenekwu, Kristina Prus, Divjot Singh Lamba, Julie Makani, Quentin Eichbaum, Luiz Amorim, Silvano Wendel, France Pirenne, Vernon Louw, Evan M. Bloch

**Affiliations:** ^1^ Department of Pathology, Microbiology and Immunology Vanderbilt University Nashville Tennessee USA; ^2^ Department of Haematology Sultan Qaboos University Hospital, University Medical City Muscat Oman; ^3^ College of Medicine and Health Sciences Sultan Qaboos University Muscat Oman; ^4^ Department of Pathology and Molecular Medicine Queen's University and Kingston Health Sciences Centre Toronto Canada; ^5^ Department of Pathology & Laboratory Medicine Children's National Hospital Washington District of Columbia USA; ^6^ Division of Transfusion Medicine, Department of Pathology Johns Hopkins University School of Medicine Baltimore Maryland USA; ^7^ International Society for Blood Transfusion Amsterdam The Netherlands; ^8^ Association for the Advancement of Blood and Biotherapies Bethesda Maryland USA; ^9^ African Society for Blood Transfusion Cairo Egypt; ^10^ Faculty of Medicine and Biomedical Sciences Université de Yaoundé Yaoundé Cameroon; ^11^ Department of Pathology and Laboratory Medicine University of Wisconsin School of Medicine and Public Health Madison Wisconsin USA; ^12^ Division of Pathology (Transfusion Medicine, Coagulation) Cincinnati Children's Hospital Medical Center Cincinnati Ohio USA; ^13^ Department of Pathology and Laboratory Medicine University of Cincinnati College of Medicine Cincinnati Ohio USA; ^14^ Department of Transfusion Medicine Post Graduate Institute of Medical Education and Research (PGIMER) Chandigarh India; ^15^ Sickle Cell Program Muhimbili University of Health and Allied Sciences Dar es Salaam Tanzania; ^16^ Department of Hematology and Blood Transfusion Muhimbili University of Health and Allied Sciences Dar es Salaam Tanzania; ^17^ Imperial Sickle Cell Group, Centre for Haematology, Department of Immunology and Inflammation Imperial College London London UK; ^18^ Roswell Park Comprehensive Cancer Center Buffalo New York USA; ^19^ HEMORIO Rio de Janeiro Brazil; ^20^ Consultant in Transfusion Medicine, Cellular Therapy and Blood Bank São Paulo Brazil; ^21^ University Paris Est Créteil INSERM U955, and Etablissement Français du Sang Créteil France; ^22^ Division of Clinical Haematology, Department of Medicine University of Cape Town and Groote Schuur Hospital Cape Town South Africa; ^23^ Division of Clinical Haematology, Department of Medicine University of Stellenbosch and Tygerberg Hospital Cape Town South Africa

## Abstract

**Background:**

Sickle cell disease (SCD) affects over 7 million people globally, with blood transfusion remaining a cornerstone of management. However, contemporary transfusion practices across diverse settings remain poorly characterized. We evaluated global transfusion practices for patients with SCD to identify gaps and inform resource prioritization.

**Study Design and Methods:**

We conducted a cross‐sectional web‐based survey of clinicians and laboratory professionals providing transfusion support for SCD, distributed via email using three professional society's membership lists (July–September 2025). Variables included facility characteristics, pre‐transfusion testing capabilities, antigen‐matching strategies, transfusion modalities, and barriers to care, stratified by World Bank income classification.

**Results:**

After excluding incomplete/duplicate responses, 102 facilities from 39 countries were analyzed; 95 actively treated patients with SCD. Facilities were predominantly public (73%), with 46% in lower‐middle‐income countries (LMICs). Routine newborn screening was performed by 33% overall (48% high‐income countries [HICs] vs. 0% low‐income). While ABO/RhD typing was nearly universal, antibody screening was available in only 72% of facilities (0% low‐income, 47% LMICs, 98% HICs). Prophylactic RBC antigen matching was performed by 46% (18% LMICs vs. 70% HICs), primarily limited to Rh(CcEe) and K. Automated RBC exchange was available in 44% overall (0% low‐income, 25% LMICs, 73% HICs); among facilities with exchange capability, 83% of HICs versus 36% of LMICs could provide exchange within 24 h for acute indications.

**Discussion:**

Pronounced income‐related disparities exist in SCD transfusion support. Facilities in lower‐income settings disproportionately lack antibody testing, prophylactic matching, and timely automated exchange. Targeted infrastructure investment and context‐appropriate guidelines are essential for equitable care.

**Abbreviations**
AABB: Association for the Advancement of Blood and Biotherapies
AfSBT: African Society for Blood Transfusion
ASH: American Society of Hematology
DHTRs: delayed hemolytic transfusion reactions
HbSS: hemoglobin S
HICs: high‐income countries
HSCT: hematopoietic stem cell transplantation
IAT: indirect antiglobulin test
ISBT: International Society of Blood Transfusion
LICs: low‐income countries
LMICs: lower‐middle‐income countries
RBC: red blood cell
RCE: RBC exchange
SCD: sickle cell disease
UMICs: upper‐middle income countries

## 1 INTRODUCTION

Globally, sickle cell disease (SCD) is the most common inherited hemoglobinopathy, affecting more than 7 million individuals, with an additional 515,000 born each year.^1^, ^2^, ^3^ SCD is largely concentrated in sub‐Saharan Africa, India, the Middle East, and among populations of African descent in the Americas and Europe.^2^, ^3^, ^4^, ^5^, ^6^, ^7^ Despite the high disease burden, many health systems—particularly in low‐ and lower‐middle‐income countries—lack the infrastructure to provide consistent, evidence‐based transfusion care for patients with SCD.^1^, ^3^, ^8^, ^9^, ^10^

SCD encompasses several genotypes, most commonly homozygous hemoglobin S (HbSS), hemoglobin SC disease, and sickle β‐thalassemia.^11^ SCD due to HbSS arises from a single point mutation in the β‐globin gene that promotes hemoglobin S polymerization under hypoxic conditions, causing red blood cell (RBC) sickling, precipitating hemolytic anemia, vaso‐occlusion, and inflammation.^12^, ^13^, ^14^ Over a lifetime, patients experience recurrent acute complications (e.g., vaso‐occlusive crises, acute chest syndrome, splenic sequestration, stroke) and chronic organ damage that frequently necessitate transfusion. In both high‐income countries (HICs) and low‐ and middle‐income countries (LMICs), transfusion is used for emergent management of severe anemia and acute complications and, in some settings, for prevention of complications, including primary and recurrent stroke.^15^, ^16^ However, the safety and effectiveness of transfusion support depend on the capacity for immunohematology testing and red cell genotyping, availability and appropriate selection of RBC components, and access to modalities such as automated RBC exchange (RCE).

Patients with SCD have disproportionately high rates of RBC alloimmunization (ranging from 5% to 47%) compared with the general transfused population (approximately 2%–5%).^17^, ^18^, ^19^, ^20^ There are several factors that contribute to this risk, including racial/ethnic disparities between predominantly African‐descent SCD populations and predominantly White blood donor pools in many countries, chronic transfusion exposure with repeated opportunities for sensitization, and enhanced immune responsiveness secondary to chronic inflammation.^20^, ^21^, ^22^, ^23^, ^24^ This high rate of alloimmunization increases the risk of acute and delayed hemolytic transfusion reactions (DHTRs) and hyperhemolysis; it also complicates procurement of compatible blood for acute and chronic transfusion and impedes hematopoietic stem cell transplantation (HSCT) and gene‐based therapies, that might otherwise prove to be curative.^23^, ^25^, ^26^, ^27^ Chronic transfusion also poses risk of iron overload (i.e., siderosis), which can lead to progressive hepatic fibrosis and cirrhosis, cardiomyopathy and heart failure, and endocrinopathies (e.g., diabetes mellitus, hypogonadism, hypothyroidism).^28^, ^29^

Transfusion strategies employed in HICs to mitigate transfusion‐related complications include prophylactic RBC antigen matching^30^, ^31^ and RCE.^32^, ^33^, ^34^ However, these strategies prove challenging in low‐resource settings given the associated need for immunohematology capabilities, equipment, and skilled personnel pertaining to blood banking and therapeutic apheresis. Consequently, most data on transfusion support in SCD overrepresent HICs.^35^, ^36^, ^37^ Understanding contemporary transfusion practices for SCD across diverse settings is needed to identify gaps in care, prioritize and advocate for resources, and support the development of locally applicable, evidence‐based transfusion guidelines.

## 2 METHODS

### 2.1 Study design and participants

We conducted a cross‐sectional, web‐based survey of clinicians and laboratory professionals who provide transfusion support for patients with SCD worldwide. Eligible respondents included physicians, nurses, transfusion medicine specialists, blood bank personnel, and other health professionals practicing in academic medical centers, community hospitals, and blood centers. The Johns Hopkins University Institutional Review Board approved the study (IRB00488304).

### 2.2 Survey instrument

The instrument was developed by the investigators in collaboration with an international panel of transfusion medicine and hematology experts with experience in SCD across multiple regions (Data S1, Supporting Information). Pilot testing was performed by six investigators from five centers in three countries to assess clarity, completeness, and relevance across resource settings. Feedback focused on simplifying terminology, removing redundant items, clarifying response options, and ensuring applicability to both hospital‐based transfusion services and blood centers. After pilot testing, the final survey comprised 35 items grouped into five domains: (1) facility characteristics (country, setting, annual SCD transfusion volume); (2) pre‐transfusion testing (ABO/RhD typing, antibody screening/identification, crossmatch); (3) prophylactic antigen‐matching policies (antigens and strategies); (4) availability of transfusion modalities (simple transfusion, manual exchange, RCE); and (5) barriers to RCE.

### 2.3 Data collection

The survey was distributed electronically via the email distribution lists for members of three major international professional organizations: Association for the Advancement of Blood and Biotherapies (AABB), the African Society for Blood Transfusion (AfSBT), and the International Society of Blood Transfusion (ISBT). The survey was open from July 11, 2025 to September 30, 2025, with two reminder emails at approximately 4‐week intervals. Participants could save their responses and return to the survey as many times as necessary. Participation was voluntary and no incentives were provided. Responses were collected using a secure web‐based platform (REDCap) hosted at Johns Hopkins University. Respondents were asked to provide the name of their institution to prevent analysis of duplicative responses, but no individual personal or identifying information (e.g., respondents' names or email addresses) was collected. All results were analyzed in aggregate.

### 2.4 Statistical analysis

We summarized data using descriptive statistics, reporting categorical variables as counts and percentages calculated among facilities with non‐missing responses for each item. To explore contextual differences, we stratified results by World Bank income classification for fiscal year 2026 (high, upper‐middle, lower‐middle, low) using the Atlas method.^38^ Given the descriptive design and non‐probability sampling, we did not perform formal hypothesis testing. Analyses were conducted in Prism version 10.6.1 (GraphPad Software, La Jolla, CA, USA).

## 3 RESULTS

### 3.1 Respondent characteristics

A total of 153 responses were received. From this initial set, 13 responses were excluded for being incomplete or lacking a location. An additional 41 responses were identified as duplicates, representing 20 unique institutions in 14 countries. For three of these institutions, duplicate responses were fully concordant and thus consolidated into a single entry for each institution in the analysis. The remaining duplicate responses from 17 institutions could not be consolidated and were excluded. This data curation process yielded a final cohort of 102 facilities from 39 countries across 5 continents (Table 1, Figures 1 and S1).

**TABLE 1:** Respondent demographics and facility characteristics.

Characteristic	Low income (*n* = 3)	Lower‐middle income (*n* = 47)	Upper‐middle income (*n* = 10)	High income (*n* = 42)	Overall (*n* = 102)
Countries represented	3	12	8	16	39
Public/government facility	2 (66.7%)	37 (78.7%)	7 (70.0%)	29 (69.0%)	75 (73.5%)
Private facility	1 (33.3%)	8 (17.0%)	3 (30.0%)	11 (26.2%)	23 (22.5%)
Other	0 (0.0%)	2 (4.3%)	0 (0.0%)	2 (4.8%)	4 (3.9%)
Geographic region					
Sub‐Saharan Africa	3 (100.0%)	35 (74.5%)	3 (30.0%)	0 (0.0%)	41 (40.2%)
Europe	0 (0.0%)	0 (0.0%)	0 (0.0%)	21 (50.0%)	21 (20.6%)
North America	0 (0.0%)	0 (0.0%)	0 (0.0%)	17 (40.5%)	17 (16.7%)
South Asia	0 (0.0%)	10 (21.3%)	0 (0.0%)	0 (0.0%)	10 (9.8%)
Middle East and North Africa	0 (0.0%)	2 (4.3%)	1 (10.0%)	4 (9.5%)	7 (6.9%)
Southeast Asia	0 (0.0%)	0 (0.0%)	3 (30.0%)	0 (0.0%)	3 (2.9%)
Latin America and Caribbean	0 (0.0%)	0 (0.0%)	3 (30.0%)	0 (0.0%)	3 (2.9%)
Age groups served					
Adults (≥18 years)	2 (66.7%)	47 (100.0%)	10 (100.0%)	40 (95.2%)	99 (97.1%)
Pediatrics (<18 years)	2 (66.7%)	42 (89.4%)	9 (90.0%)	30 (71.4%)	83 (81.4%)
Neonates	0 (0.0%)	35 (74.5%)	9 (90.0%)	27 (64.3%)	71 (69.6%)

*Note*: Numbers represent the number of facilities in each world bank category.

**FIGURE 1:**
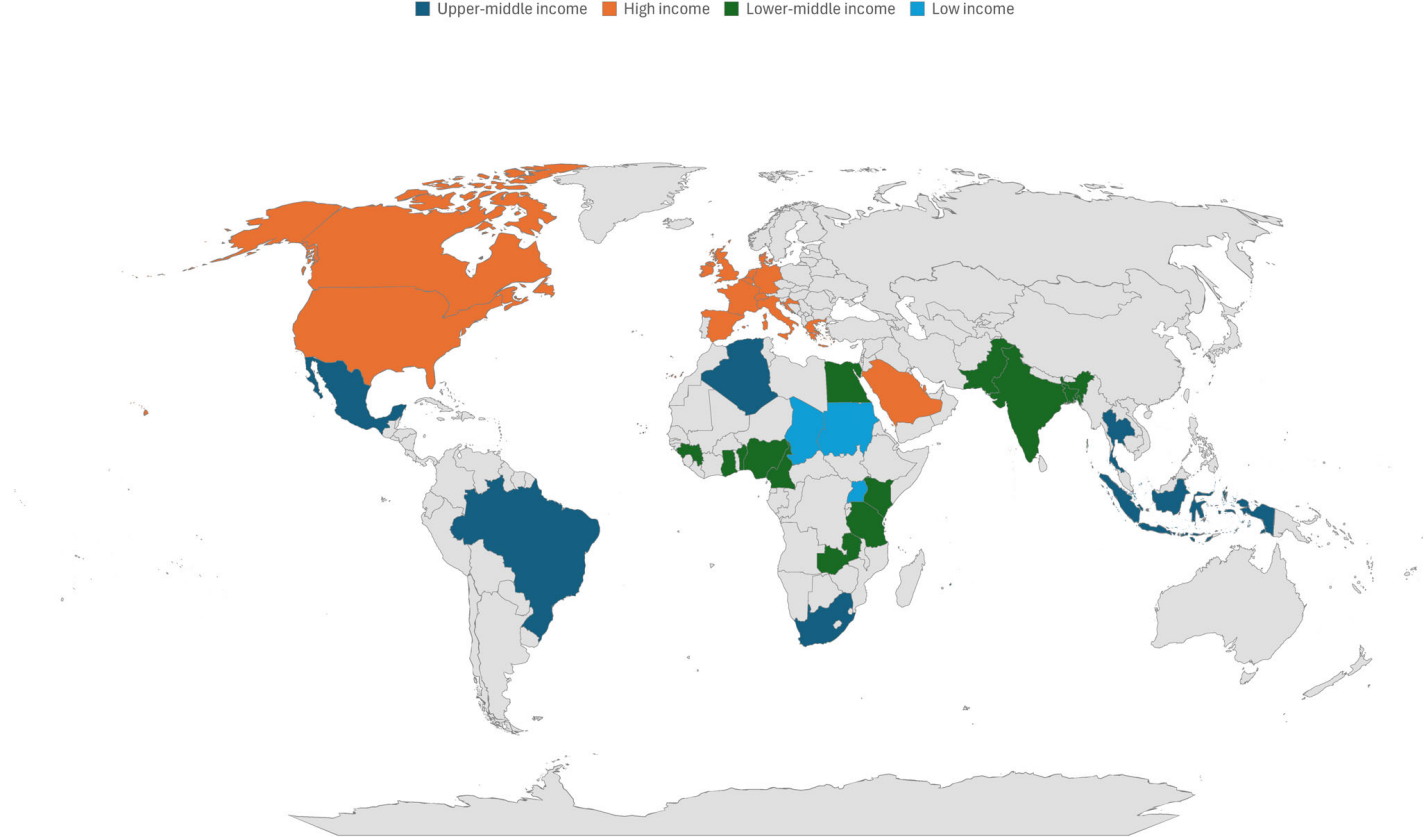
Country of respondents by World Bank income classification.

The 102 respondents represented 7 geographic regions, with the highest representation from sub‐Saharan Africa (40.2%), Europe (20.6%), and North America (16.7%). The five countries with the most participating facilities were the United States and Nigeria (13 each), Ghana (9), India and Italy (6 each). The distribution by World Bank income classification was: 3 (2.9%) facilities from 3 low‐income countries (LICs), 47 (46.1%) facilities from 12 LMICs, 10 (9.8%) facilities from 8 upper‐middle income countries (UMICs), and 42 (41.2%) facilities from 16 HICs (Table S1). The majority of facilities were public or government‐run (73.5%), with nearly all facilities caring for adult patients (97.1%) and most caring for pediatric patients (81.4%) and neonates (69.6%) (Table 1).

Among all 102 facilities, 7 (6.9%) reported treating zero patients with SCD annually (3 facilities from 2 LMICs, 2 facilities from 1 UMIC, 2 facilities from 2 HICs); consequently, these facilities were excluded from analyses of transfusion management practices for patients with SCD (e.g., prophylactic antigen matching, transfusion procedures), as they could not meaningfully report on routine practices without the relevant patient population. However, they were included in analyses of infrastructure and laboratory capabilities (e.g., testing equipment availability, blood sourcing methods) because their existing infrastructure reflects potential capacity for future SCD services. Therefore, infrastructure‐based analyses included all 102 facilities, while practice‐based analyses utilized data from 95 facilities actively treating SCD patients.

The volume of patients with SCD treated annually varied widely among the 95 facilities that reported caring for this population. The most common category was 1–50 unique patients treated annually (38.9%), followed by 51–100 patients (16.8%), and more than 500 patients (13.7%).

### 3.2 Neonatal screening and initial assessment practices

Routine neonatal SCD screening was performed at birth in 32.6% of facilities who answered the question (*n* = 92), with variation by income status: 0% in LICs, 22.0% in LMICs, 25.0% in UMICs, and 47.5% in HICs (Table 2). Reflex RBC antigen genotyping for newborns with SCD was rare overall (31.0% of all facilities with screening who responded), with 7 of the 9 (77.8%) facilities located in HICs.

**TABLE 2:** Neonatal screening and initial assessment practices by income status.

Practice	Low income (*n* = 3)	Lower‐middle income (*n* = 47)	Upper‐middle income (*n* = 10)	High income (*n* = 42)	Overall (*n* = 102)
Routine SCD screening at birth^a^	0/3 (0.0%)	9/41 (22.0%)	2/8 (25.0%)	19/40 (47.5%)	30/92 (32.6%)
Reflex RBC genotyping for newborns with SCD^b^	0/0 (0.0%)	2/9 (22.2%)	0/2 (0.0%)	7/18 (38.9%)	9/29 (31.0%)
At first encounter with SCD patient					
RBC antigen phenotype	0/3 (0.0%)	9/44 (20.5%)	6/8 (75.0%)	32/40 (80.0%)	47/95 (49.5%)
RBC antigen genotype	1/3 (33.3%)	6/44 (13.6%)	2/8 (25.0%)	17/40 (42.5%)	26/95 (27.4%)
Neither phenotype nor genotype	2/3 (66.7%)	30/44 (68.2%)	1/8 (12.5%)	4/40 (10.0%)	37/95 (38.9%)
Central registry of alloantibodies/genotype^c^	0/3 (0.0%)	7/45 (15.6%)	3/10 (30.0%)	17/41 (41.5%)	27/99 (27.3%)

^a^ Neonatal screening data based on 92 facilities (10 missing: 6 lower‐middle income, 2 upper‐middle income, 2 high income).

^b^ Reflex genotyping calculated among 29 facilities with neonatal screening programs who responded (1 high‐income facility with missing data excluded).

^c^ Central registry data based on 99 facilities (3 missing: 2 lower‐middle income, 1 high income).

At the first patient encounter, RBC antigen phenotyping was performed in 49.5% of facilities that treat patients with SCD, while RBC antigen genotyping at first encounter was less common at 27.4% overall, primarily in HICs and UMICs. However, RBC genotyping was available at seven of the facilities in LICs and LMICs (India, Ghana, Nigeria, Uganda, Zambia). Notably, 37/95 (38.9%) of facilities did not perform phenotyping or genotyping at first encounter, with most of these facilities located in LMICs and UMICs, although four facilities in HICs did not report these capabilities.

A central registry of alloantibodies and/or genotype/phenotype information was reported by 27.3% of facilities who responded, including 41.5% of facilities in HICs, 30.0% of facilities in UMICs, and 15.6% of facilities in LMICs; there were no facilities in LICs with a central registry.

### 3.3 Blood product sourcing

Blood sourcing practices varied by income status (Table 3). Onsite blood banks were most common overall (63.7%), particularly in LMICs (78.7%) and UMICs (90.0%), while HICs more frequently utilized regional blood centers (54.8%). LICs were more likely to use a combination of onsite, regional, and national blood centers. On demand, or walking blood banks, was uncommon (5.9% overall).

**TABLE 3:** Blood product sourcing and donor testing by income status.

Blood sourcing/testing	Low income (*n* = 3)	Lower‐middle income (*n* = 47)	Upper‐middle income (*n* = 10)	High income (*n* = 42)	Overall (*n* = 102)
Blood sourcing (select all that apply)					
Onsite blood bank	1 (33.3%)	37 (78.7%)	9 (90.0%)	18 (42.9%)	65 (63.7%)
Regional blood center	1 (33.3%)	12 (25.5%)	4 (40.0%)	23 (54.8%)	40 (39.2%)
National blood center	1 (33.3%)	10 (21.3%)	3 (30.0%)	14 (33.3%)	28 (27.5%)
Private blood center	0 (0.0%)	6 (12.8%)	0 (0.0%)	3 (7.1%)	9 (8.8%)
On‐demand/walking blood bank^a^	0 (0.0%)	3 (6.4%)	1 (10.0%)	2 (4.8%)	6 (5.9%)
Testing donor units for hemoglobin S (*n* = 2 low income, *n* = 36 lower‐middle income, *n* = 8 upper‐middle income, *n* = 39 high income, *n* = 85 overall)^b^					
Always test	0 (0.0%)	9 (25.0%)	1 (12.5%)	18 (46.2%)	28 (32.9%)
Sometimes test	0 (0.0%)	8 (22.2%)	0 (0.0%)	2 (5.1%)	10 (11.8%)
Never test	2 (100%)	19 (52.8%)	7 (87.5%)	19 (48.7%)	47 (55.3%)

^a^ On‐demand/walking blood bank refers to local donor collection performed in response to immediate clinical need, rather than routine sourcing from an established onsite, regional, or national blood inventory.

^b^ Hemoglobin S testing data based on 85 facilities who treat SCD patients and responded (17 facilities excluded: 1 low income, 11 lower‐middle income, 2 upper‐middle income, 3 high income). Blood sourcing data based on all 102 facilities.

Testing of donor blood units for hemoglobin S was performed by 44.7% of facilities who answered the question (*n* = 85). This practice was more common in HICs (46.2% always, 5.1% sometimes) and LMICs (25.0% always, 22.2% sometimes) but was rarely performed (12.5% always, 0% sometimes) in UMICs and absent in LICs.

### 3.4 Pre‐transfusion testing practices and methodologies

While ABO/RhD typing and crossmatching were near‐universal, antibody screening was available in only 71.6% of facilities (0% LICs, 46.8% LMICs, 100% UMICs, 97.6% HICs) (Table 4). Antibody identification was less available (57.8% overall), particularly in low and lower‐middle‐income countries.

**TABLE 4:** Pre‐transfusion testing capabilities by income status.

Testing capability	Low income (*n* = 3)	Lower‐middle income (*n* = 47)	Upper‐middle income (*n* = 10)	High income (*n* = 42)	Overall (*n* = 102)
Recipient testing available					
ABO typing	3 (100.0%)	43 (91.5%)	10 (100.0%)	41 (97.6%)	97 (95.1%)
RhD typing	3 (100.0%)	42 (89.4%)	10 (100.0%)	40 (95.2%)	95 (93.1%)
Antibody screen	0 (0.0%)	22 (46.8%)	10 (100.0%)	41 (97.6%)	73 (71.6%)
Antibody identification	0 (0.0%)	12 (25.5%)	9 (90.0%)	38 (90.5%)	59 (57.8%)
Crossmatch	3 (100.0%)	42 (89.4%)	10 (100.0%)	40 (95.2%)	95 (93.1%)

*Note*: Testing capabilities based on all 102 facilities. Note: values below 100% may reflect a true response, missing responses, facility‐level variation, referral‐based testing models, or respondent uncertainty regarding services performed by external blood suppliers/reference laboratories.

Testing methodologies varied by income status (Table S2). For ABO and RhD typing, tile testing (slide method) was common in lower‐income settings (66.7% low‐income, 53.2% lower‐middle‐income), with 9.3% of facilities relying exclusively on this methodology, while automated column agglutination predominated in higher‐income countries. Tube testing was used across all income levels (58.8% overall for ABO, 56.9% for RhD). For antibody screening, automated column agglutination was most common, particularly in UMICs and HICs. Crossmatching was most frequently performed by the indirect antiglobulin test (IAT), while electronic crossmatch was available almost exclusively in high‐income countries with minimal use elsewhere.

### 3.5 Facilities treating patients with SCD

#### 3.5.1 Red blood cell antigen matching strategies

Among the 95 facilities that treat patients with SCD, 90 provided data on matching and 41 (45.6%) of these performed prophylactic RBC antigen matching: 0/3 (0%) low‐income, 7/39 (17.9%) lower‐middle income, 6/8 (75.0%) upper‐middle income, and 28/40 (70.0%) high‐income facilities (Table 5). Among facilities performing prophylactic matching, the antigens most commonly matched other than ABO and RhD were C, c, E, e, and K. Additional matching for Fy^a^, Fy^b^, Jk^a^, Jk^b^, S, and s was less common and primarily performed in UMICs and HICs.

**TABLE 5:** Antigen matching strategies by matching type and income status.

	Low income	Lower‐middle income	Upper‐middle income	High income	Overall
Prophylactic matching (no antibody formed)—calculated among facilities treating SCD patients with data					
Facilities performing prophylactic matching	0/3 (0%)	7/39 (17.9%)	6/8 (75.0%)	28/40 (70.0%)	41/90 (45.6%)
Antigens matched prophylactically (among *n* = 41 facilities performing prophylactic matching)					
D	N/A	5/7 (71.4%)	6/6 (100%)	28/28 (100%)	39/41 (95.1%)
C/c	N/A	1/7 (14.3%)	6/6 (100%)	28/28 (100%)	35/41 (85.4%)
E/e	N/A	1/7 (14.3%)	6/6 (100%)	28/28 (100%)	35/41 (85.4%)
K	N/A	1/7 (14.3%)	6/6 (100%)	28/28 (100%)	35/41 (85.4%)
Duffy (Fy^a^, Fy^b^)	N/A	0/7 (0.0%)	4/6 (66.7%)	10/28 (35.7%)	14/41 (34.1%)
Kidd (Jk^a^, Jk^b^)	N/A	0/7 (0.0%)	4/6 (66.7%)	12/28 (42.9%)	16/41 (39.0%)
S/s	N/A	0/7 (0.0%)	3/6 (50.0%)	12/28 (42.9%)	15/41 (36.6%)
Prospective matching (after antibody identified)—calculated among facilities treating SCD patients with antibody screening capability and data: *n* = 0 low income, *n* = 11 lower‐middle income, *n* = 8 upper‐middle income, *n* = 37 high income, *n* = 56 overall					
Facilities performing prospective matching	0/0 (0%)	8/11 (72.7%)	7/8 (87.5%)	36/37 (97.3%)	51/56 (91.1%)
Matching strategy used (among facilities performing prospective matching: *n* = 0 low income, *n* = 8 lower‐middle income, *n* = 7 upper‐middle income, *n* = 36 high‐income, *n* = 51 overall)					
Extended matched units (C, c, E, e, K, Fy^a^, Fy^b^, Jk^a^, Jk^b^, S, s) plus antibody‐specific	N/A	1/8 (12.5%)	3/7 (42.9%)	21/36 (58.3%)	25/51 (49.0%)
C, c, E, e and K plus antibody‐specific	N/A	3/8 (37.5%)	2/7 (28.6%)	8/36 (22.2%)	13/51 (25.5%)
Antibody‐specific only	N/A	4/8 (50.0%)	2/7 (28.6%)	7/36 (19.4%)	13/51 (25.5%)

*Note*: Denominators for each section are specified in the particular section. For prophylactic matching, percentages are calculated among all facilities in each income group who provided data (five facilities in four lower‐middle‐income countries did not respond). For prospective matching, percentages are calculated among facilities with antibody screening capability only.

Among 56 facilities that treat patients with SCD with antibody screening capability and data on prospective matching, 51 (91.1%) performed prospective antigen matching after antibody identification. Among facilities performing prospective matching, the most common strategy was to provide extended matched units in addition to the antigen to which the recipient has an antibody (49.0% overall; 12.5% lower‐middle income, 42.9% upper‐middle income, 58.3% high‐income). Matching only for Rh and K in addition to the antibody‐specific antigen was the primary strategy in LMICs and was reported in 22.2% of HICs. Notably, 25.5% of facilities provided units that were negative only for the antibody‐specific antigen, including in 19.4% of HICs. Among the 13 facilities that only match for the cognate antigen, 7 nonetheless still perform limited or extended RBC antigen phenotyping/genotyping.

### 3.6 Transfusion procedures and modalities

Simple RBC transfusion was the most widely available modality (89.5% overall), with similar availability across income groups (Table 6). Simple whole blood transfusion was common in LICs (100%) and LMICs (50.0%) but was rare in UMICs (0.0%) and HICs (2.5%). Manual RBC exchange was available in 37.9% of facilities, while RCE was offered by 44.2% of facilities, though this technology was primarily available in HICs (72.5%), with only one‐quarter of UMICs and LMICs, and no LICs reporting access. The primary barrier to automated apheresis was equipment cost and availability, particularly in lower‐middle income countries. RBC‐only products were predominantly used in high‐income facilities, while facilities in LMICs showed more varied practice with substantial use of both RBC and whole blood products. Low‐income facilities were evenly split between RBC only, whole blood only, and both.

**TABLE 6:** Transfusion procedures and barriers by income status.

Procedure/barrier	Low income	Lower‐middle income	Upper‐middle income	High income	Overall
Transfusion procedures offered					
Simple RBC transfusion	2/3 (66.7%)	38/44 (86.4%)	8/8 (100.0%)	37/40 (92.5%)	85/95 (89.5%)
Simple whole blood transfusion	3/3 (100%)	22/44 (50.0%)	0/0 (0.0%)	1/40 (2.5%)	26/95 (27.4%)
Manual RBC exchange	1/3 (33.3%)	10/44 (22.7%)	5/8 (62.5%)	20/40 (50.0%)	36/95 (37.9%)
Automated RBC exchange (apheresis)	0/3 (0.0%)	11/44 (25.0%)	2/8 (25.0%)	29/40 (72.5%)	42/95 (44.2%)
Blood product selection for transfusion					
Red blood cells only	1/3 (33.3%)	22/40 (55.0%)	7/8 (87.5%)	39/40 (97.5%)	69/91 (75.8%)
Both red blood cells and whole blood	1/3 (33.3%)	17/40 (42.5%)	1/8 (12.5%)	0/40 (0.0%)	19/91 (20.9%)
Whole blood only	1/3 (33.3%)	1/40 (2.5%)	0/8 (0.0%)	1/40 (2.5%)	3/91 (3.3%)
Barriers to automated apheresis (among facilities without automated exchange)					
Equipment cost/availability	2/3 (66.7%)	26/33 (78.8%)	4/6 (66.7%)	5/11 (45.5%)	37/53 (69.8%)
Lack of trained staff	0/3 (0.0%)	12/33 (36.4%)	1/6 (16.7%)	6/11 (54.5%)	19/53 (35.8%)
Difficulty accessing blood products	1/3 (33.3%)	10/33 (30.3%)	0/6 (0.0%)	0/11 (0.0%)	11/53 (20.8%)
Lack of institutional support	0/3 (0.0%)	12/33 (36.4%)	2/6 (33.3%)	3/11 (27.3%)	17/53 (32.1%)

*Note*: Transfusion procedures calculated among 95 facilities treating SCD patients. Blood product selection excludes four facilities with missing/not sure responses (*n* = 91). Barriers calculated among 53 facilities treating SCD patients without automated exchange capability. Values below 100% may reflect true responses, missing responses, facility‐level variation, referral‐based testing models, or respondent uncertainty regarding services performed by external blood suppliers/reference laboratories.

### 3.7 Indications for automated red blood cell exchange

Among the 42 facilities offering RCE, the most common indications were acute stroke or transient ischemic attack, severe acute chest syndrome requiring oxygen, and secondary stroke prevention (Table S3).

For acute indications (e.g., stroke), the majority of facilities (71.4%) could provide RCE within hours (i.e., <24 h) (Table S4). However, 82.8% of high‐income facilities could provide exchange within hours compared to only 36.4% of lower‐middle income facilities. Among facilities in LMICs, 36.4% required 1–3 days and 9.1% required longer than 7 days to provide exchange.

## 4 DISCUSSION

This global survey of transfusion practices for patients with SCD highlights pronounced disparities in access to comprehensive immunohematology services and advanced transfusion modalities. Respondents in LICs and LMICs, particularly in sub‐Saharan Africa and parts of Asia where the burden of SCD is greatest, reported substantial barriers to accessing evidence‐based practice, such as newborn screening for SCD, immunohematology testing, and related laboratory support. Practices varied widely across settings, impacting both transfusion safety and clinical outcomes.

Pre‐transfusion testing capacity varied markedly by country income status, as documented previously.^39^ Countries bearing the greatest burden of SCD also tended to have more fragmented blood services and limited immunohematology infrastructure.^8^, ^10^, ^39^, ^40^ While ABO/RhD typing and crossmatching were nearly universal, more advanced testing was uncommon in low‐ and lower‐middle‐income settings, whereby antibody identification capacity was limited, and antibody screening was absent in all low‐income facilities. These gaps impact the implementation of American Society of Hematology (ASH) guidance, which recommends obtaining an extended red cell antigen profile (by genotype or serology) for all patients with SCD at the earliest opportunity—ideally before the first transfusion—rather than relying on ABO/RhD typing alone.^17^ Yet more than two‐thirds of facilities in LICs and LMICs did not perform baseline RBC phenotyping or genotyping, and approximately 80% did not routinely perform newborn SCD screening, limiting early diagnosis and pre‐transfusion planning. Important gaps persisted even in HICs, as 10% of facilities do not perform baseline phenotyping or genotyping and fewer than half reported neonatal screening or had access to a centralized registry of alloantibodies or patient genotypes.

There was heterogeneity in the provision of HbS‐negative (sickle trait‐negative) RBCs for patients with SCD. While testing was most common in HICs, it was absent in LICs, and rare in many UMICs. HbS‐negative blood is typically selected to avoid transfusing HbS‐containing units and to ensure accurate HbS% quantification in chronic transfusion or exchange programs.^11^, ^41^ Moreover, some well‐resourced systems have limited or discontinued routine HbS unit screening, in part due to leukoreduction filter failures with sickle trait donations.^42^ HbS screening may be relevant in regions with higher sickle trait prevalence and limited leukoreduction; however, testing capacity remains limited in lower‐income settings, despite the availability of inexpensive screening methods (e.g., HbS solubility test).

Nearly half of facilities lacked capacity for antibody screening by IAT. Without routine screening, alloantibodies will be missed, risking acute or DHTR that can precipitate life‐threatening hemolysis and rarely, hyperhemolysis.^27^, ^43^, ^44^, ^45^ Automated column agglutination technology, which has become widely used in HICs, improves standardization and sensitivity. By contrast, most LIC and LMIC facilities rely on manual tube or tile (slide) testing for ABO and RhD typing. Tile testing, while inexpensive, has a lower sensitivity than automated or manual tube testing and may fail to detect weak RhD variants or mixed‐field reactions.^46^ When used at room temperature, it will likely not detect clinically significant IgG antibodies that react optimally at 37°C by IAT.^47^ Even facilities with some capacity for antibody screening may not be adequately equipped to manage alloimmunization in patients with SCD. Commercially available three‐cell screening panels may miss a third of clinically significant alloantibodies in patients with SCD, particularly antibodies against antigens that are more prevalent in African populations.^43^ Addressing this requires developing screening panels incorporating RBCs expressing antigens that may be rare in Western European populations, but more common in Africans, such as MNS6 (He), MNS25 (Dantu), and KEL6 (Jsa).^42^

Even in HICs, there is substantial heterogeneity in RBC antigen‐matching practices. Prophylactic matching for Rh and K antigens reduces the rates of alloimmunization in patients with SCD,^48^, ^49^ prompting its recommendation for management of SCD.^17^, ^50^ However, limited prophylactic RBC antigen matching and even extended prophylactic antigen matching do not eliminate alloimmunization risk entirely.^30^ Prophylactic antigen matching for patients without alloantibodies was performed by fewer than half of facilities, and in 70% of the facilities in HICs. Where prophylaxis was used, matching primarily centered on Rh and K, consistent with international guidelines.^50^

At the time of writing, international guidance indicates that there is insufficient evidence to support transfusion with RBCs prophylactically matched beyond ABO, RhD, RhCcEe, and K to reduce the risk of alloimmunization or DHTRs in patients with SCD who are already alloimmunized.^50^ Nevertheless, almost half of all facilities with antibody screening capability performed extended prospective matching (beyond Rh and K) for patients who developed alloantibodies, whereas approximately one‐quarter matched only for Rh and K, in addition to the antigen to which the alloantibody is directed. These patterns likely reflect challenges pertaining to inventory management, access to typing reagents, and cold chain infrastructure,^10^ as well as an evidence‐based emphasis on the most immunogenic antigens (RhCcEe, K), with broader matching adopted selectively where resources allow.^17^, ^50^

None of the facilities in LICs and only a quarter of facilities in LMICs reported access to RCE. By contrast, almost three quarters of facilities in HICs reported RCE capability. Further, even when available, almost two‐thirds of lower‐middle income facilities were unable to perform RCE for acute indications (e.g., stroke) within hours when it would be most useful to prevent deterioration.^51^, ^52^ Barriers cited by respondents included the cost and availability of apheresis equipment, shortages of trained personnel, and inconsistent access to appropriate blood components. Some facilities were unable to deploy timely exchange for acute indications, despite being in possession of the necessary apheresis equipment.

Table 7 proposes goals that can be modified according to local resources and SCD burden. Within this framework, a short‐term priority in many settings is strengthening newborn screening and building core immunohematology capacity, including routine baseline phenotyping (or genotyping where feasible), prophylactic matching for Rh and K, and reliable antibody screening and identification, supported by adoption of more sensitive and standardized testing methods (e.g., tube or column agglutination rather than tile testing). In many settings the limiting factor is not only equipment but also funding systems, supply chains, and staffing. Targeted subsidies, reagent and equipment‐sharing models, and local reagent production can lower costs and stabilize supply, and workforce development (training, competency assessment, continuing education) should proceed alongside the adoption of quality systems and accreditation pathways. International transfusion organizations, including the ISBT, AABB, and AfSBT, can support this work through harmonized, resource‐stratified guidelines, educational programming tailored to low‐resource and non‐Anglophone settings, and accreditation programs. Finally, expanding access to red blood cell exchange requires addressing both equipment and systems barriers.^8^, ^9^ Regional models or shared capital programs may enable facilities to use apheresis technology without each buying its own, supported by tele‐mentored training and expert networks such as the ISBT Clinical Transfusion Working Party hemoglobinopathy subgroup. This group could connect facilities in LICs and LMICs with international expertise and reference testing capacity for complex alloantibody investigation. Where timely RCE cannot be reliably delivered for acute indications, standardizing and ensuring universal access to manual exchange may provide greater short‐term clinical impact while longer‐term systems are developed.

**TABLE 7:** Resource‐stratified goals for transfusion support in SCD.

Domain	Low income	Lower‐middle income	Upper‐middle income	High income
Early identification	Establish reliable diagnosis pathway (targeted screening where universal newborn screening not yet feasible) + linkage to care	Phased newborn screening (regional → national) + linkage to care	Routine newborn screening with defined follow‐up	Universal newborn screening + robust linkage to comprehensive care
Core pretransfusion testing	Reliable ABO/RhD typing + crossmatch; implement IAT antibody screen as first “step‐up” goal (onsite or referral)	Routine IAT antibody screen + defined pathway for antibody identification (onsite or reference lab)	Routine antibody screen + antibody identification; competency + QC	Comprehensive antibody testing with rapid ID and complex workups; strong QC/QA
Baseline antigen profile	Baseline phenotype at earliest feasible encounter (at least RhD, RhCcEe and K when possible) for patients likely to be repeatedly transfused	Routine baseline phenotype (or genotype where feasible) early in care	Routine baseline phenotype/genotype; strategies for Rh variants	Genotype‐informed care pathways; broad Rh variant capability
Prophylactic antigen matching	Prioritize Rh (C/c, E/e) and K matching for chronically transfused/high‐risk patients as inventory allows	Routine Rh (C/c, E/e) and K matching for SCD patients receiving ongoing transfusion	Expand matching for selected patients (e.g., alloimmunized) where feasible	Maintain robust antigen‐typed inventory; individualized matching strategies when indicated
Testing methodology	Transition from tile‐only methods toward tube/IAT workflows; standard operating procedures + basic QC	Expand standardized tube/IAT; introduce column agglutination where feasible	Broad adoption of column agglutination and/or solid phase, with or without automation	Fully standardized column agglutination/solid phase platforms + external quality assessment
Component selection	Define an SCD component policy (e.g., antigen‐matched when possible; inventory protections for chronically transfused patients)	Strengthen component specifications for SCD programs; plan for HbS‐negative access where feasible (via supplier or local testing)	Routine access to HbS‐negative components for SCD when indicated	Reliable HbS‐negative provisioning per local policy; mature inventory management and traceability
Exchange capability	Standardize manual exchange protocols for acute indications; establish referral pathway for automated exchange when possible	Manual exchange broadly available; develop regional automated exchange hubs	Automated exchange available with defined on‐call pathways	Automated exchange widely available with rapid‐response capability for acute indications
Information systems	Written transfusion protocols; basic tracking of antibodies/adverse reactions	Local registry/record‐sharing strategy (even if paper‐based); structured hemovigilance	Electronic registries and decision support where feasible	Integrated registries (alloantibodies/genotypes) + electronic decision support + mature hemovigilance
Workforce and quality systems	Core staff training + competency assessment; implement basic QA	Scale training + mentorship; pursue AfSBT/AABB/ISBT stepwise quality frameworks	Expand accreditation and external quality assessment	Full quality systems and accreditation with continuous improvement cycles

Abbreviations: AABB, Association for the Advancement of Blood and Biotherapies; AfSBT, African Society for Blood Transfusion; IAT, indirect antiglobulin test; ISBT, International Society of Blood Transfusion; QA, quality assurance; QC, quality control; SCD, sickle cell disease.

This study has several limitations. First, the survey was distributed through professional organization listservs, and administered only in English, which likely biased participation toward better‐resourced centers and more engaged practitioners. Consequently, our findings likely overestimate the availability of transfusion services and immunohematology capacity, particularly in resource‐limited settings and non‐Anglophone countries. Second, responses from LICs were limited, whereas LMICs (including a substantial representation from sub‐Saharan Africa) comprised a larger share of respondents; thus, our LMIC estimates may more strongly reflect lower‐middle‐income settings than the lowest‐resource environments. This response pattern also suggests that transfusion services in LICs may be less connected to professional organizations and quality frameworks captured by our sampling approach. Third, data were self‐reported and not independently verified, introducing potential misclassification, recall error, and social desirability bias, particularly if respondents were unfamiliar with referral testing pathways (e.g., reference‐laboratory send‐outs or blood supplier practices). Additionally, the number of responses from some countries and regions was small (e.g., South America), limiting precision and precluding formal statistical comparisons. Likewise, in countries represented by only one or a small number of facilities, including countries with centralized or national hemoglobinopathy programs, responses should be interpreted as facility‐level observations rather than nationally representative estimates. The survey also did not collect certain population data or practices such as donor pool HbS trait prevalence, leukoreduction practices, or donor selection policies, limiting our ability to define setting‐specific thresholds for donor unit HbS screening. Finally, the survey emphasized structural capacity (availability, policies) rather than process measures (adherence) or outcomes, and the cross‐sectional design provides only a snapshot of current practice and cannot assess trends or the effects of recent policy changes.

Despite these limitations, this global survey highlights substantial income‐based disparities in transfusion practices for patients with SCD. Access to newborn screening, RBC antigen phenotyping, comprehensive antibody testing, prophylactic antigen‐matching, and RCE remains largely restricted to higher‐resource settings, while the countries with the greatest SCD burden often lack even basic immunohematology infrastructure. Equipment availability, trained personnel, and governmental support represent key modifiable barriers to optimizing transfusion care in LICs and LMICs where the greatest burden of SCD is encountered. Addressing these gaps through targeted capacity‐building initiatives, policy advocacy, and international collaboration is essential to achieving equitable, high‐quality transfusion support for all patients with SCD worldwide.

## CONFLICT OF INTEREST STATEMENT

Evan M. Bloch reports personal fees and non‐financial support from Terumo BCT, Grifols, Abbott Laboratories, and UptoDate, outside of the submitted work. Evan M. Bloch is a member of the United States Food and Drug Administration (FDA) Blood Products Advisory Committee. Any views or opinions that are expressed in this manuscript are those of the author's, based on his own scientific expertise and professional judgment; they do not necessarily represent the views of either the Blood Products Advisory Committee or the formal position of FDA, and do not bind or otherwise obligate or commit either Advisory Committee or the Agency to the views expressed. Jeannie Callum reports funding support for clinical trials from Canadian Blood Services and Octapharma, and speaking fees from Octapharma and Pfizer. Vernon Louw reports receiving fees as a non‐executive board member of the Western Cape Blood Service, and also receives conference travel and accommodation support as a Board Member of NATA.

## Supporting information


**FIGURE S1.** Diagram of survey participant flow.
**TABLE S1.** World Bank income classification of respondent countries.
**TABLE S2.** Pre‐transfusion testing methodologies by income status (among facilities performing each test and not specific for patients with sickle cell disease).
**TABLE S2.** Indications for automated red blood cell exchange by World Bank income status.
**TABLE S3.** Time to obtain automated red blood cell exchange for acute indications by World Bank income status.

## Data Availability

The data that support the findings of this study are available from the corresponding author upon reasonable request.
