# Supplementary material for: A global survey of blood transfusion practices for patients with sickle cell disease
Source: Transfusion. 2026 Jul 15;66(10):1926–38. doi: 10.1111/trf.70331 (PMC13674188; doi:10.1111/trf.70331)
Supplement: Supplementary file 1 [file TRF-66-1926-s001.docx]

**A global survey of blood transfusion practices for patients with sickle cell disease**

**Authors:** Jeremy W. Jacobs,^1^ Arwa Z. Al-Riyami,^2,3^ Jeannie Callum,^4^ Meghan Delaney,^5^ David Daniel,^6^ Jenny White,^7^ Christopher Bocquet,^8^ Mohammed Farouk,^9^ Claude Tayou,^10^ Chinelo P. Onyenekwu,^11^ Kristina Prus,^12,13^ Divjot Singh Lamba,^14^ Julie Makani,^15,16,17^ Quentin Eichbaum,^18^ Luiz Amorim,^19^ Silvano Wendel,^20^ France Pirenne,^21^ Vernon Louw,^22,23^ Evan M. Bloch^6^

^1^ Department of Pathology, Microbiology and Immunology, Vanderbilt University, Nashville, TN, USA

^2^ Department of Haematology, Sultan Qaboos University Hospital, University Medical City, Muscat, Oman

^3^ College of Medicine and Health Sciences, Sultan Qaboos University, Muscat, Oman

^4^ Department of Pathology and Molecular Medicine, Queen's University and Kingston Health Sciences Centre, Toronto, Canada

^5^ Department of Pathology & Laboratory Medicine, Children's National Hospital, Washington, DC, USA

^6^ Division of Transfusion Medicine, Department of Pathology, Johns Hopkins University School of Medicine, Baltimore, MD, USA

^7^ International Society for Blood Transfusion, Amsterdam, The Netherlands

^8^ Association for the Advancement of Blood and Biotherapies, Bethesda, MD, USA

^9^ African Society for Blood Transfusion, Cairo, Egypt

^10^ Faculty of Medicine and Biomedical Sciences, Université de Yaoundé, Yaoundé, Cameroon

^11^ Department of Pathology and Laboratory Medicine, University of Wisconsin School of Medicine and Public Health, Madison, WI, USA

^12^ Division of Pathology (Transfusion Medicine, Coagulation), Cincinnati Children's Hospital Medical Center, Cincinnati, OH, USA

^13^ Department of Pathology and Laboratory Medicine, University of Cincinnati College of Medicine, Cincinnati, OH, USA

^14^ Department of Transfusion Medicine, Post Graduate Institute of Medical Education and Research (PGIMER), Chandigarh, India

^15^ Sickle Cell Program, Muhimbili University of Health and Allied Sciences, Dar es Salaam, Tanzania

^16^ Department of Hematology and Blood Transfusion, Muhimbili University of Health and Allied Sciences, Dar es Salaam, Tanzania

^17^ Imperial Sickle Cell Group, Centre for Haematology, Department of Immunology and Inflammation, Imperial College London

^18^ Roswell Park Comprehensive Cancer Center, Buffalo, NY, USA

^19^ HEMORIO, HEMORIO, Rio de Janeiro, Brazil

^20^ Hospital Sirio-Libanês Blood Bank, São Paulo, Brazil

^21^ University Paris Est Créteil, INSERM U955, and Etablissement Français du Sang, Créteil, France

^22^ Division of Clinical Haematology, Department of Medicine, University of Cape Town and Groote Schuur Hospital, Cape Town, South Africa

^23^ Division of Clinical Haematology, Department of Medicine, University of Stellenbosch and Tygerberg Hospital, Cape Town, South Africa

Corresponding author: Jeremy W. Jacobs, MD, MHS

1211 Medical Center Drive

Nashville, TN 37211

[Jeremy.w.jacobs@vumc.org](mailto:Jeremy.w.jacobs@vumc.org)

**Table of Contents:**

**Page 2:** Supplemental Figure 1. Diagram of survey participant flow.

**Pages 3-4:** Supplemental Table 1. World Bank income classification of respondent countries

**Page 5:** Supplemental Table 2. Pre-transfusion testing methodologies by income status (among facilities performing each test and not specific for patients with sickle cell disease)

**Page 6:** Supplemental Table 2. Indications for automated red blood cell exchange by World Bank income status

**Page 7:** Supplemental Table 3. Time to obtain automated red blood cell Exchange for acute indications by World Bank income status

**Supplemental Figure 1.** Survey participant flow

Of 153 responses received, 13 were excluded for being incomplete or lacking a location. Among the remaining responses, 41 duplicate responses from 20 institutions were identified; concordant duplicates from 3 institutions were consolidated into a single entry each, and discordant duplicate responses from 17 institutions were excluded. The final cohort comprised 102 facilities from 39 countries across 5 continents. Of these, 95 actively treated patients with SCD and contributed to practice-based analyses, while 7 reported no SCD patients and were included only in infrastructure and laboratory capability analyses.

**Supplemental Table 1.** World Bank income classification of respondent countries

| **Country** | **Income Status** | **Number of responding facilities** |
| --- | --- | --- |
| Chad | Low income | 1 |
| Sudan | Low income | 1 |
| Uganda | Low income | 1 |
| Bangladesh | Lower-middle income | 1 |
| Bénin | Lower-middle income | 1 |
| Cameroon | Lower-middle income | 2 |
| Egypt | Lower-middle income | 2 |
| Ghana | Lower-middle income | 9 |
| Guinea | Lower-middle income | 1 |
| India | Lower-middle income | 6 |
| Kenya | Lower-middle income | 2 |
| Nigeria | Lower-middle income | 13 |
| Pakistan | Lower-middle income | 3 |
| Tanzania | Lower-middle income | 5 |
| Zambia | Lower-middle income | 2 |
| Algeria | Upper-middle income | 1 |
| Brazil | Upper-middle income | 1 |
| Cabo Verde | Upper-middle income | 1 |
| Indonesia | Upper-middle income | 1 |
| Mauritius | Upper-middle income | 1 |
| Mexico | Upper-middle income | 2 |
| South Africa | Upper-middle income | 1 |
| Thailand | Upper-middle income | 2 |
| Belgium | High income | 1 |
| Canada | High income | 4 |
| Croatia | High income | 1 |
| Denmark | High income | 1 |
| France | High income | 1 |
| Germany | High income | 1 |
| Greece | High income | 2 |
| Ireland | High income | 1 |
| Italy | High income | 6 |
| Qatar | High income | 2 |
| Saudi Arabia | High income | 2 |
| Spain | High income | 2 |
| Switzerland | High income | 2 |
| The Netherlands | High income | 2 |
| United Kingdom | High income | 1 |
| USA | High income | 13 |

**Supplemental Table 2.** Pre-transfusion testing methodologies by income status (among facilities performing each test and not specific for patients with sickle cell disease)

| **Testing Methodology** | **Low income** | **Lower-middle income** | **Upper-middle income** | **High income** | **Overall** |
| --- | --- | --- | --- | --- | --- |
| **ABO/RhD typing methods**** | **(n=3)*** | **(n=43)*** | **(n=10)*** | **(n=41)*** | **(n=97)*** |
| Tile testing | 2 (66.7%) | 25 (58.1%) | 2 (20.0%) | 3 (7.3%) | 32 (33.0%) |
| Tube testing | 1 (33.3%) | 28 (65.1%) | 7 (70.0%) | 24 (58.5%) | 60 (61.9%) |
| Manual column agglutination | 0 (0.0%) | 11 (25.6%) | 7 (70.0%) | 16 (39.0%) | 34 (35.1%) |
| Automated column agglutination | 0 (0.0%) | 9 (20.9%) | 8 (80.0%) | 33 (80.5%) | 50 (51.5%) |
| Automated solid phase | 0 (0.0%) | 6 (14.0%) | 0 (0.0%) | 10 (24.4%) | 16 (16.5%) |
| **Antibody screening methods**** | **(n=0)†** | **(n=22)†** | **(n=10)†** | **(n=41)†** | **(n=73)†** |
| Tube testing | - | 13 (59.1%) | 4 (40.0%) | 19 (46.3%) | 36 (49.3%) |
| Manual column agglutination | - | 11 (50.0%) | 7 (70.0%) | 17 (41.5%) | 35 (47.9%) |
| Automated column agglutination | - | 6 (27.3%) | 8 (80.0%) | 32 (78.0%) | 46 (63.0%) |
| Automated solid phase | - | 3 (13.6%) | 0 (0.0%) | 10 (24.4%) | 13 (17.8%) |
| **Crossmatch methods**** | **(n=3)‡** | **(n=42)‡** | **(n=10)‡** | **(n=40)‡** | **(n=95)‡** |
| Electronic crossmatch | 0 (0.0%) | 0 (0.0%) | 2 (20.0%) | 23 (57.5%) | 25 (26.3%) |
| Immediate spin crossmatch | 1 (33.3%) | 24 (57.1%) | 6 (60.0%) | 19 (47.5%) | 50 (52.6%) |
| Antihuman globulin crossmatch | 2 (66.7%) | 31 (73.8%) | 9 (90.0%) | 37 (92.5%) | 79 (83.2%) |

**Among facilities performing ABO/RhD typing. †Among facilities performing antibody screening (no low-income facilities had this capability). ‡Among facilities performing crossmatch.*

***More than one method could be selected.*

**Supplemental Table 3.** Indications for automated red blood cell exchange by World Bank income status

| **Indication** | **Lower-middle income (n=11)** | **Upper-middle income (n=2)** | **High income (n=29)** | **Overall (n=42)** |
| --- | --- | --- | --- | --- |
| Acute stroke/TIA | 9 (81.8%) | 2 (100%) | 21 (72.4%) | 32 (76.2%) |
| Secondary stroke prevention | 4 (36.4%) | 2 (100%) | 19 (65.5%) | 25 (59.5%) |
| Severe ACS (oxygen required) | 5 (45.5%) | 2 (100%) | 19 (65.5%) | 26 (61.9%) |
| Preparation for general anesthesia | 1 (9.1%) | 2 (100%) | 19 (65.5%) | 22 (52.4%) |
| Acute vaso-occlusive pain crisis | 5 (45.5%) | 1 (50.0%) | 14 (48.3%) | 20 (47.6%) |
| Recurrent pain crises | 5 (45.5%) | 1 (50.0%) | 14 (48.3%) | 20 (47.6%) |
| Priapism | 5 (45.5%) | 1 (50.0%) | 13 (44.8%) | 19 (45.2%) |
| Sickle hepatopathy | 4 (36.4%) | 2 (100%) | 12 (41.4%) | 18 (42.9%) |
| ACS prevention | 1 (9.1%) | 2 (100%) | 12 (41.4%) | 15 (35.7%) |
| Pregnancy | 1 (9.1%) | 2 (100%) | 12 (41.4%) | 15 (35.7%) |

*Note: Percentages calculated among facilities offering automated red blood cell exchange (n=42; 0 low income, 11 lower-middle income, 2 upper-middle income, 29 high income).*

**Supplemental Table 4.** Time to obtain automated red blood cell Exchange for acute indications by World Bank income status

| **Time to Exchange** | **Lower-middle income (n=11)** | **Upper-middle income (n=2)** | **High income (n=29)** | **Overall (n=42)** |
| --- | --- | --- | --- | --- |
| Within hours | 4 (36.4%) | 2 (100%) | 24 (82.8%) | 30 (71.4%) |
| 1-3 days | 4 (36.4%) | 0 (0.0%) | 3 (10.3%) | 7 (16.7%) |
| Longer than 7 days | 1 (9.1%) | 0 (0.0%) | 0 (0.0%) | 1 (2.4%) |
| Other | 0 (0.0%) | 0 (0.0%) | 1 (3.4%) | 1 (2.4%) |
| Missing data | 2 (18.2%) | 0 (0.0%) | 1 (3.4%) | 3 (7.1%) |

*Note: Percentages calculated among facilities offering automated red blood cell exchange (n=42; 0 low income, 11 lower-middle income, 2 upper-middle income, 29 high income).*
